# BDSM: pathological or healthy expression of intimacy?

**DOI:** 10.1192/j.eurpsy.2022.2083

**Published:** 2022-09-01

**Authors:** E. Wuyts

**Affiliations:** Collaborative Antwerp Psychiatric Research Institute (CAPRI), Faculty Of Medicine And Health Sciences, Campus Drie Eiken, University Of Antwerp, Edegem, Belgium

**Keywords:** BDSM, stigma, Sexual health, Biological mechanisms

## Abstract

**Introduction:**

Though BDSM interest (bondage & discipline, dominance & submission and sadism & masochism) has proven to be quite prevalent (46.8% in recent research), there is still significant stigma surrounding it, both in general society and among mental health practitioners.

**Objectives:**

This research explores the biological mechanisms associated with a BDSM interaction in the hope to strengthen the argument that it does not belong in the psychiatric field.

**Methods:**

The present study collected data on peripheral hormone levels, pain thresholds and pain cognitions before and after a BDSM interaction and compared these results to a control group.

**Results:**

show that submissives have increased cortisol and endocannabinoid levels due to the BDSM interaction and that these increases are linked. Dominants showed a significant increase in endocannabinoids associated with power play but not with pain play. BDSM practitioners have a higher pain threshold overall and a BSDM interaction will result in a temporary elevation of pain thresholds for submissives. Additionally, pain thresholds in dominants will be dependent upon their fear of pain and tendency to catastrophize pain and submissives will experience less fear of pain than the control group

**Conclusions:**

Even though this is one of the first studies of its kind, several biological processes can be associated with BDSM interactions, strengthening the hypothesis of BDSM as a healthy form of intimacy and promoting its distinction from paraphilias as they are described in the DSM or ICD classifications.

**Disclosure:**

No significant relationships.

